# A stem group *Codium* alga from the latest Ediacaran of South China provides taxonomic insight into the early diversification of the plant kingdom

**DOI:** 10.1186/s12915-022-01394-0

**Published:** 2022-09-21

**Authors:** Shu Chai, Cédric Aria, Hong Hua

**Affiliations:** 1 Shaanxi Key Laboratory of Early Life and Environments, Northwest University, Xi’an 710069, People’s Republic of China; 2 Present address: Department of Ecology and Evolutionary Biology, University of Toronto, 25 Willcocks Street, Toronto, ON M5S 3B2 Canada; 3 Present address: Department of Natural History, Royal Ontario Museum, 100 Queen’s Park, Toronto, ON M5S 2C6 Canada

**Keywords:** Ediacaran, Chlorophyta, *Codium*, Evolution, Dengying Formation

## Abstract

**Background:**

In recent years, Precambrian lifeforms have generated an ever-increasing interest because they revealed a rich eukaryotic diversity prior to the Cambrian explosion of modern animals. Among them, macroalgae are known to be a conspicuous component of Neoproterozoic ecosystems, and chlorophytes in particular are already documented in the Tonian, when they were so far expected to originate. However, like for other major eukaryotic lineages, and despite predictions of molecular clock analyses placing roots of these lineages well into the Neoproterozoic, a taxonomic constraint on Precambrian green algae has remained difficult.

**Results:**

Here, we present an exceptionally preserved spherical, coenocytic unicellular alga from the latest Ediacaran Dengying Formation of South China (> ca. 541 Ma), known from both external and internal morphology, fully tridimensional and in great detail. Tomographic X-ray and electronic microscopy revealed a characteristic medulla made of intertwined siphons and tightly packed peripheral utricles, suggesting these fossils belong to the Bryopsidales genus *Codium*. However, its distinctly smaller size compared to extant species leads us to create *Protocodium sinense* gen. et sp. nov. and a phylomorphospace investigation points to a possible stem group affinity.

**Conclusions:**

Our finding has several important implications. First, *Protocodium* allows for a more precise calibration of Archaeplastida and directly confirms that a group as derived as Ulvophyceae was already well diversified in various ecosystems prior to the Cambrian explosion. Details of tridimensional morphology also invite a reassessment of the identification of other Ediacaran algae, such as *Chuaria*, to better discriminate mono-versus multicellularity, and suggest unicellular *Codium*-like morphotypes could be much older and widespread. More broadly, *Protocodium* provides insights into the early diversification of the plant kingdom, the composition of Precambrian ecosystems, and the extreme longevity of certain eukaryotic plans of organization.

**Supplementary Information:**

The online version contains supplementary material available at 10.1186/s12915-022-01394-0.

## Background

Macroalgae—that is, macroscopic photosynthetic eukaryotes including many green, red, and brown algae [1]—are being documented as an increasingly conspicuous component of Precambrian marine ecosystems. According to Bykova and colleagues [1], 359 pre-Cambrian genera have been recorded so far, with 188 in the Ediacaran alone. Archaeplastid algae in particular—commonly known as red and green algae—already have a strong presence by at least the Tonian [2–5], from which can be inferred extensive modifications of benthic habitats and oceans’ near-shore biochemistry at that time, based on the known importance of these primary producers today [6, 7]. Remarkable among green algae in particular is the diversity of cellular arrangements, encompassing variations of both mono- and multicellularity, and the tremendous success of extant siphonous (multinucleate, or coenocytic) taxa, such as Bryopsidales [8]. So far, the paleontological information has been in accordance with the predictions of molecular clock studies, placing the origin of Chlorophyta at about 1 billion years [7, 9, 10], although molecular markers also suggest a delayed radiation during the Cryogenian–Ediacaran transition [6].

Nevertheless, the accuracy of this correspondence between rates of genetic evolution and the algal fossil record is also dependent on the precision of taxonomic assignments of extinct taxa. Owing to difficulties in accessing important diagnostic characters such as internal structure, biochemistry, or life cycles, systematic work has often consisted in creating fossil taxa independent of the extant classification of archaeplastids [11, 12]. While the Ediacaran “Doushantuo” material—otherwise well-known for providing insights into a cryptic Ediacaran diversity of derived eukaryotes arguably including some metazoans [13, 14]—and the Mesoproterozoic fossil *Bangiomorpha pubescens* have provided some constraint on red algae affinities with extant lineages [4, 5, 15], the classification of Precambrian potential chlorophytes has been more tentative. *Proterocladus* Butterfield from the Svanbergfjellet Formation has been formally classified as a siphonocladalean Ulvophyceae [2, 16], and a large number of fossil green algae from the Neoproterozoic have been regarded as Ulvophyceae [3, 15, 17]—notably *Beltanelliformis brunsae*, which has been interpreted by some as a structure similar to the gametophyte of the Bryopsidales *Derbesia* [12]—but most chlorophyte taxa remain in a floating state of taxonomy, in part due to the lack of information on internal microstructures or even fine analyses of surface morphology [18].

## Results

### Primary description

**Fig. 1:**
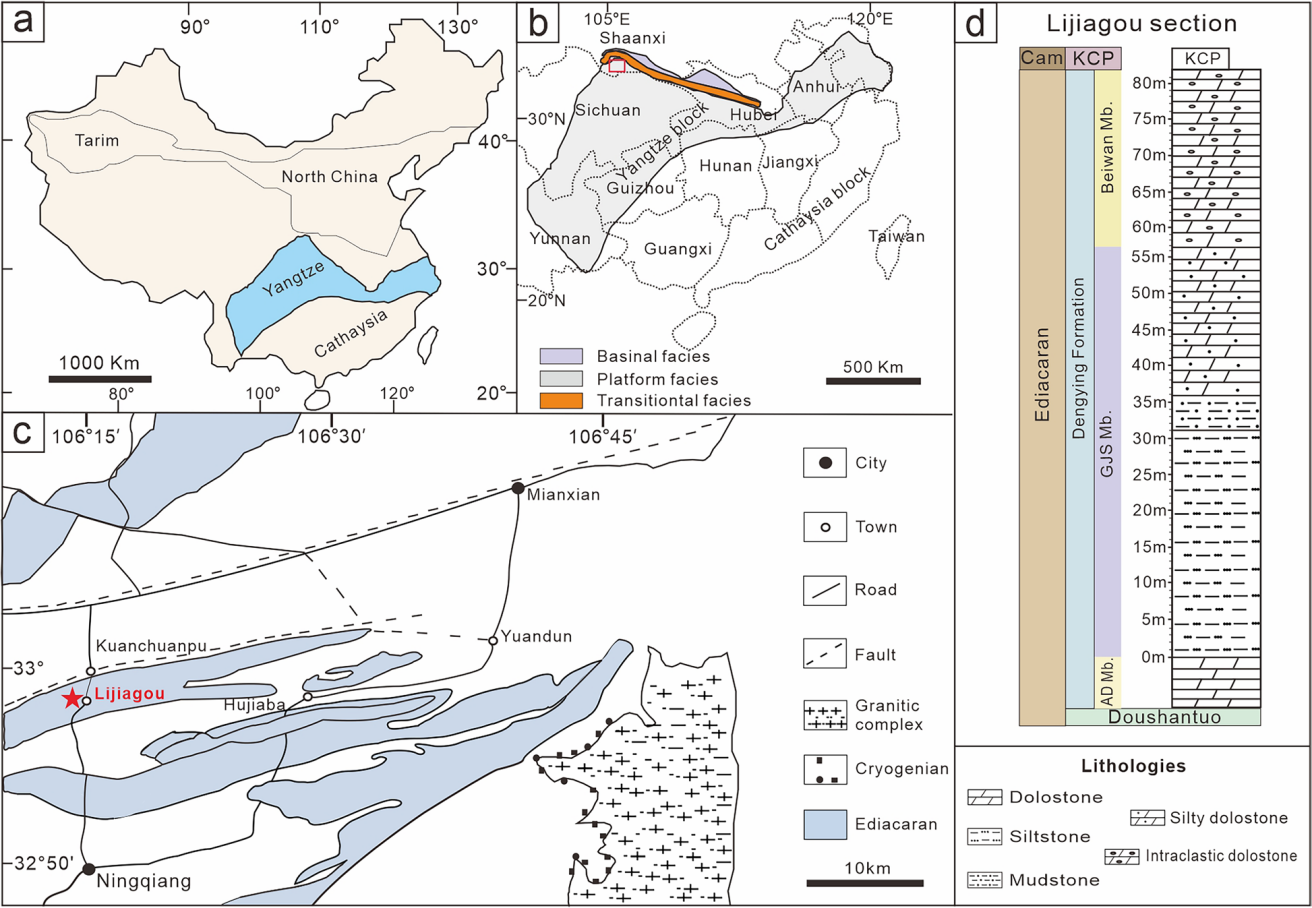
Maps and lithostratigraphy of the terminal Ediacaran Dengying Formation in the Lijiagou (LJG) area, South China. **a**, **b** Ediacaran platform depositional environments in the northwestern margin of the Yangtze Platform [20] with a red rectangle showing (**b**) the location of the Ningqiang area in **c**. **c** Geological map of the LJG area in southern Shaanxi Province. Red star points to the studied section at Lijiagou village, Ningqiang, southern Shaanxi Province. **d** Stratigraphic occurrence of the Dengying Formation at the LJG section (modified from Cai et al. [21])

Specimens presented here are from the latest Ediacaran (**> **ca. 541 Ma) Gaojiashan biota of the Dengying Formation, southern Shaanxi Province, South China (Fig. 1), well known for documenting the radiation of skeletal tubular fossils, such as *Cloudina* and *Sinotubulites* [19], but with little comparative work on diversified, well-preserved spherical fossils assemblages, in particular with respect to their possible affinities with animal embryos or algae.

**Fig. 2:**
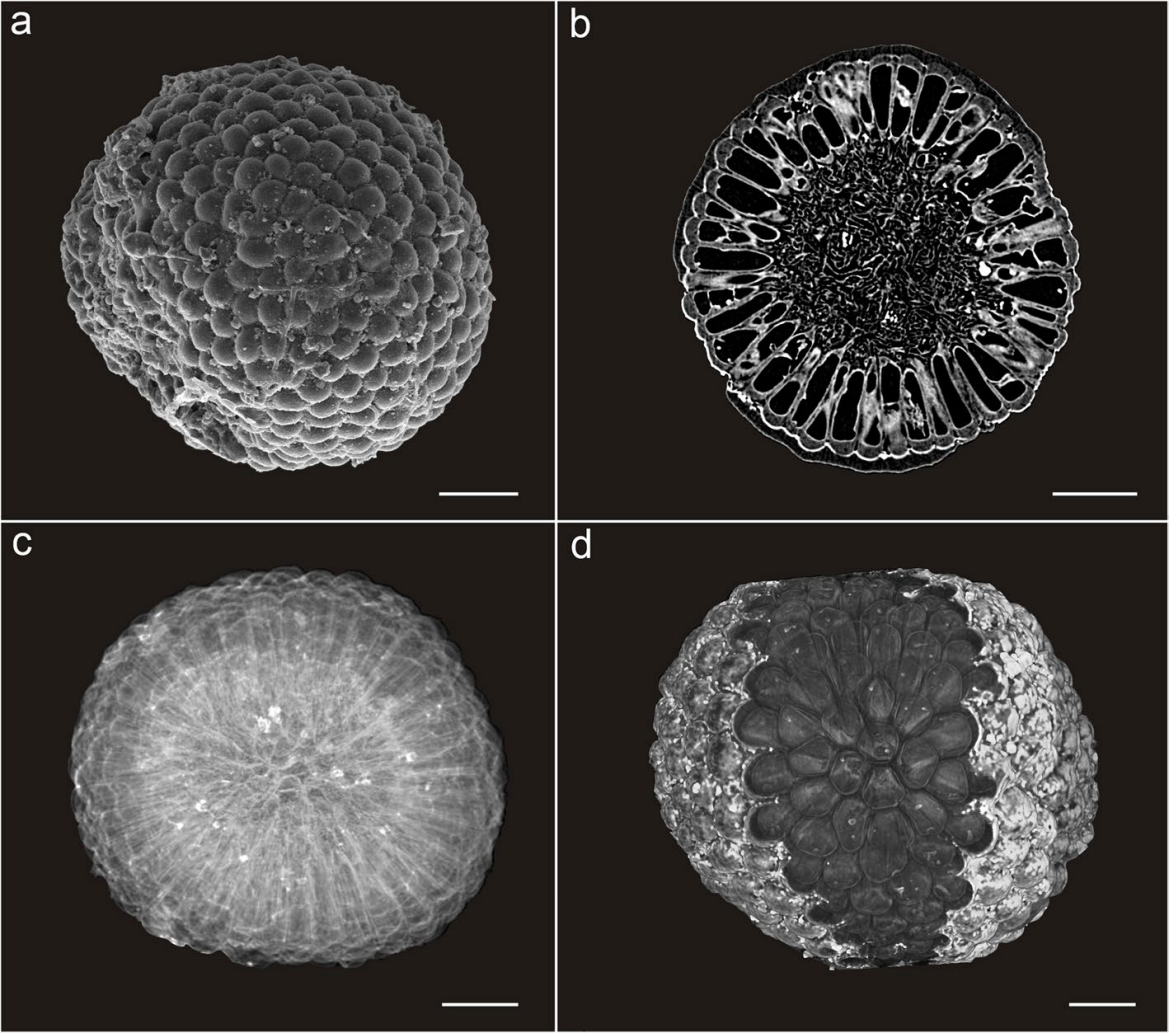
Overall morphoanatomy of *Protocodium sinense* from the Dengying Formation of South China. **a**, **c**, **d** NWULJG 10,034. **a** Overview of scanning electron microscopy (SEM) images. **b–d** Micro-CT images. **b** NWULJG 10,026, cross section view of thallus structure, showing tightly packed and clavate to cylindrical utricles and internal siphonous matrix. **c** Full view, showing both surface and inner part of thallus. **d** Full view, showing both the surface and apical internal morphology of utricles. Scale bars, 100 μm

**Fig. 3:**
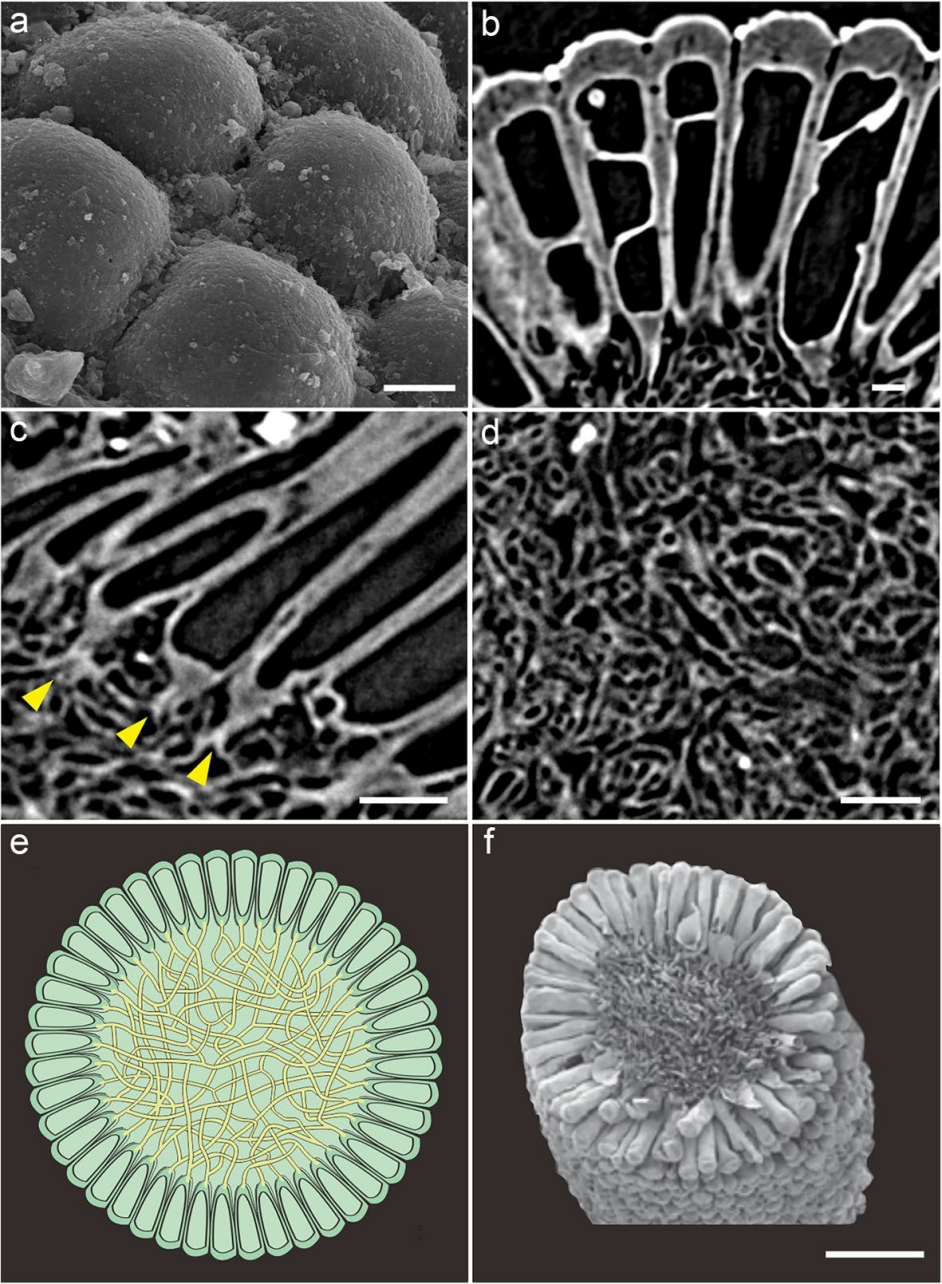
Detailed anatomy of *Protocodium sinense* and comparison with modern *Codium*. **a** NWULJG 10,034, SEM close-up of thallus surface, showing contacts between utricle tips. **b**–**d** NWULJG 10,026. **b** Tomographic close-up in cross section, showing utricles are tightly packed and clavate to cylindrical, with tips rounded and smooth. **c** Tomographic close-up in cross section, showing siphonous branching at base of utricles. Yellow solid arrows point to bifurcations of the main basal siphon. **d** Close-up of medullar siphons. **e** Diagrammatic reconstruction of *Protocodium sinense*. **f** SEM imaging of a cross section of an extant *Codium fragile *ssp*. tomentosoides* thallus (reproduced with permission from Jim Provan). Scale bars: **a** 20 μm, **b** 10 μm, **c** 50 μm, **d** 20 μm, **f** 500 μm

The five specimens are spherical, 506.76 ± 34.22 μm in diameter (Fig. 2, Additional file 1: Dataset S1, Additional file 2: Fig. S1). These spheres are composed of an outer layer of tightly juxtaposed, club-shaped tubes with rounded tips 112.00 ± 11.60 μm in length and connected via a central mass of intertwined filaments 261.76 ± 29.18 μm in width (Figs. 2 and 3). Tomographic sections show crossing lines and vesicle-shaped structures inside the tubes (Fig. 3b). The small, intertwined filaments of the inner medullar mesh show notable variations in their shape and size (average width 3.06 ± 1.13 μm; Fig. 3d, Additional file 1: Dataset S1). In the peripheral layer, the differentiated reproductive finger-like swells (Figs. 2 and 3a–e) are tightly packed and clavate to cylindrical, with tips rounded and smooth, lacking hairs (Figs. 2 and 3a–c). The external tube tip morphology is consistent within and across both specimens, and the contact between these tips is roughly pentagonal to hexagonal (Figs. 2 and 3a). At their base, tubes connect to the central mesh via a single medial filament branching basally into two other filaments (Fig. 3c, e). Fossils are preserved via phosphatization (Additional file 3: Fig. S2), but the original composition of the organic material could not be resolved.

### Interpretation

Tomographic renderings showing multiple layers (Fig. 2c, d) confirm the integrity of the tubes as undivided spaces connected at their base to a filamentous mesh. Outer-layer cells of fossilized metazoan embryos can superficially resemble our fossils [13], but the regular juxtaposition of tube tips, the constant unit number across our specimens, and details of the internal organization in *Protocodium* all rule out an embryonic identity. Although individual nuclei are not preserved, the connection of the outer layer structures to an undivided filamentous core suggests coenocytic unicellularity. No sexual structures such as gametangia are identifiable.

The irregular lines inside the tubes are artifacts of the computed tomography caused by slight differences of alignment of the tubes onto the transversal plane, which sometimes reveal part of the tubes’ wall membrane or even the cavity of overlapping tubes. Single tomographic slices show a continuous variation of this artifact, with apparent tube deformations corresponding to the superimposition of several tubes (Figs. 2b and 3b). By comparison, fossilized septa expectedly display regular width, alignment and position distinct from irregular and partial preservations of cell walls (e.g., [22]).

We therefore identify our fossil specimens as single, spherical, coenocytic siphonous cells (Fig. 2, Additional file 2: Fig. S1), as they are found specifically in bryopsidales green algae, without a larger branching or mat-shaped thallus.

### Affinity

The presence of a medullar core of small intertwined siphonous tubes surrounded by a uniform layer of elongate bulb-shaped structures is diagnostic of the genus *Codium* among siphonous green algae [23]. Our fossils are virtually indistinguishable from modern *Codium* representatives in both external and internal structure (Fig. 3e, f). A comparable organization is found in the bryopsidales relative *Halimeda* (e.g., [24]), but, to our knowledge, no member in this genus typically forms spherical thalli; in addition, in *Halimeda* utricles are short and bulbous, and siphons are stout, each branch bearing several utricles. The species is also not a dasyclad or cyclocrinitid, otherwise well-represented in the fossil record, including some problematic forms in the early Cambrian [25]. Despite some structural similarities, thalli of these algae possess a holdfast, which is an extension of the small siphonous core, while a large part of the volume remains hollow, because the outer layer is composed of small bulbous heads borne by well-spaced-out, thin primary branches [26].

However, the size of siphons and utricles in our specimen are almost an order of magnitude smaller than the average in *Codium* [27] and is also about half the size of medullar siphons and utricles of smaller individuals known within this genus, including in the well-known and invasive *C. fragile* [28]. Resolving further the affinity of our fossil species within the genus *Codium* based on morphology is made difficult by a challenging trait-based systematics confronted by considerable intraspecific variations [28] and wide-reaching convergences [23], thus still relying on identification keys [29], as well as by the significance among extant species of characters here either inapplicable, unseen, or absent (e.g., gametangia, thallus branches and utricle hairs). Morphology alone is otherwise largely insufficient to constrain phylogenetic relationships in this group [23].

**Fig. 4:**
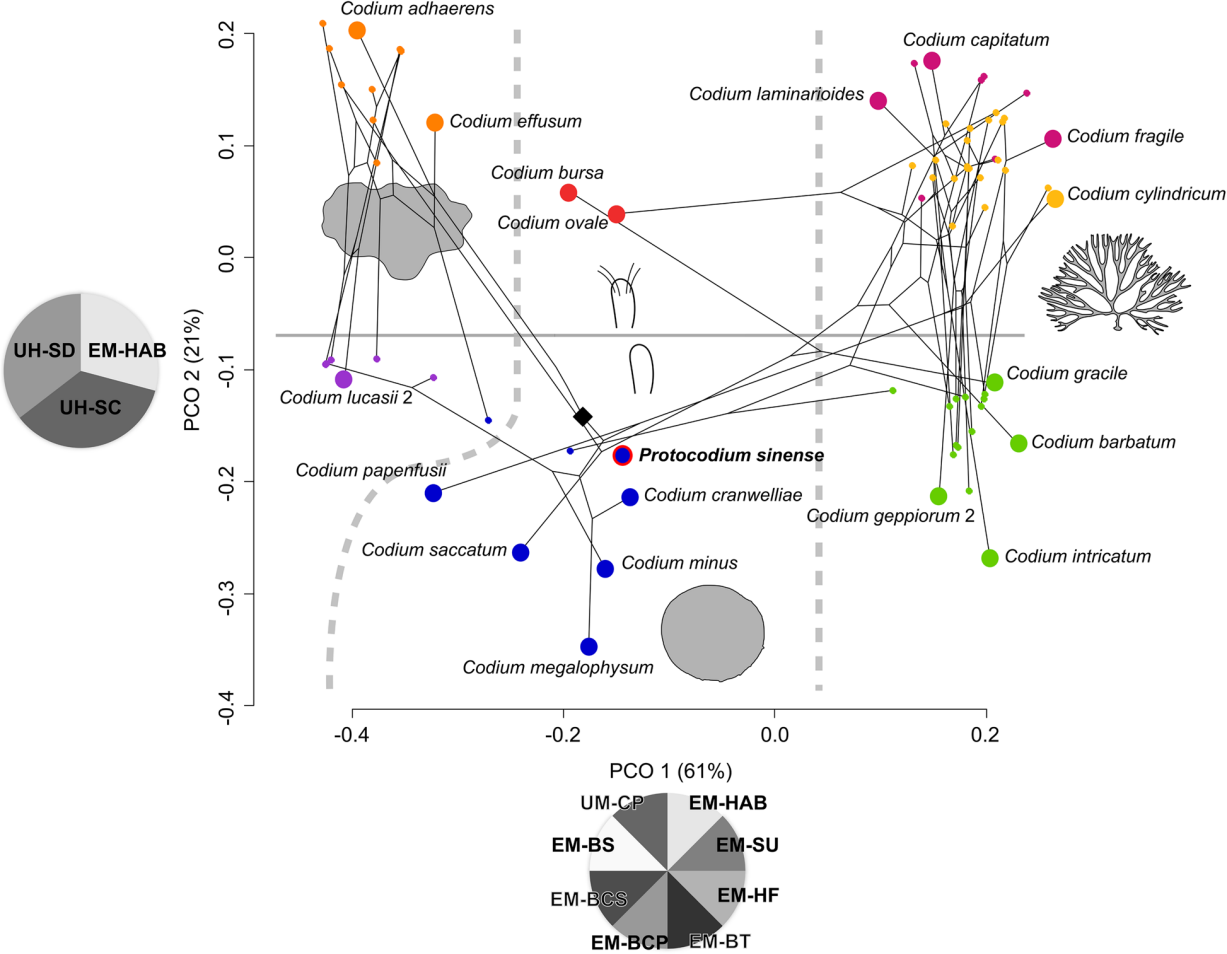
Phylomorphospace of the genus *Codium* (*n* = 72), with placement of *Protocodium sinense*. Larger circles are selected representative species. Color coding reflects K-means clustering for seven groups. Black diamond points to the root of the tree; Pie charts represent significant loadings (see Methods and Additional file 1), with acronyms taken from Verbruggen et al. [23]: EM-BCP, branch compression; EM-BCS, branch constriction; EM-BS, branch shape; EM-BT, branching type; EM-HAB, thallus habit; EM-HF, holdfast type; EM-SU, thallus surface; UH-SC, utricle hairs, presence of scars or hairs; UH-SD, utricle hairs, density; UM-CP, utricle composition. Diagrams represent the major subdivisions of the first two dimensions: thallus shape on axis 1 (left to right: mat-forming, spherical, erect/repent; 61% total variance explained) and presence (upper half)/absence (lower half) of hairs or scars on axis 2 (21% total variance explained)

For this reason, we undertook a phenetic approach integrated to molecular results for extant species instead (Fig. 4), with the intent to estimate the morphological distance between our fossils and comparable *Codium* morphogroups—specifically *C. bursa*, *C. minus*, and their relatives. Among hierarchical clustering methods, weighted pair group method with arithmetic mean (WPGMA) is the ordination closest to the principal coordinate analysis (PCOA) (Additional file 4: Fig. S3), whose axis 1 is influenced by thallus habit (mat-forming, spherical, erect, repent) and traits further describing branching patterns (Fig. 4). Although thallus type is also significant in explaining the variance of axis 2, this dimension is most conspicuously ordinated by the presence and type of utricle hairs, distinctly separating upper and lower part of the two-dimensional graph (Fig. 4). The phylomorphospace shows that these axes are also broadly consistent with high-level phylogeny, insofar as thallus habit is reflected in the separation between the main clades, especially branching versus non-branching forms [23] (Fig. 4). K-means clustering does not converge on a significant solution, but a sub-division into seven optimal clusters closely matches the PCOA ordination on axes 1 and 2 and hence major clades.

*C. bursa* resolves in its own separate cluster with *C. ovale*, while our fossils resolve in a cluster including *C. minus* and other taxa with spherical thalli (Fig. 4). The ordination therefore suggests that, based on the set of characters used to set apart different *Codium* species, our fossils are well nested within the *C. minus* cluster of spherical taxa, as opposed to lying apart from all other *Codium* species. It follows that the morphology of *Protocodium* is generally consistent with the rest of the genus and particularly the *C. minus* subgroup.

Based on these results, as well as the difference in size (by a factor of at least 2 compared to extant species) and the presence of short, single medullar connections at the base of utricles, we propose that our fossil species constitutes a stem taxon of the *Codium* genus. The lack of gametangia may be explained by the fact that our fossils are sexually immature gametophytes, which would seem more likely than to invoke a very different form of germination. The presence of a short single medullar filament at the base of utricles is not documented among spherical forms [30] but is known to be a possible developmental feature of certain mat-forming taxa, such as *C. picturatum* (H. Verbruggen, pers. comm.). As a fundamental difference, most mat-forming species possess composite utricles, with some exceptions, such as *C. coralloides* [23]. Interestingly, the thallus of mat-forming is broadly irregular but also commonly produces spherical extensions. There is therefore arguably in our fossil taxon a heterogeneous combination of traits, which associates morphologies of both spherical and mat-forming lineages.

Hence, we devise the systematic paleontology as follows:

### Systematic paleontology

Chlorophyta Reichenbach, 1828.

Ulvophyceae Stewart & Mattox, 1978.

Bryopsidales J.H. Schaffner, 1922.

Codiaceae Kützing, 1843.

*Protocodium sinense* gen. et sp. nov.

(Figs. 2 and 3, Supp. Figures 2 and 3).

LSID: urn:lsid:zoobank.org:act:397AB8FD-143C-481B-A9B7-F6F034A2E4E7.

*Material.* Holotype NWULJG 10,034; Paratypes NWULJG 10,015, 10,021, 10,026, 10,042.

*Occurrence*. Beiwan Member, Dengying Formation at the Lijiagou section in southern Shaanxi Province, China.

*Derivation of name*. From the Greek *πρῶτος*, “first”, owing to the arguably ancestral nature of the type compared to other modern *Codium* algae. The species is named after the discovery of the fossil in China.

*Diagnosis*. Cells (thalli) 506.76 ± 34.22 μm in diameter, with siphonous medulla 261.76 ± 29.18 μm and length of utricles 112.00 ± 11.60 μm.

*Description*. Individuals are single spherical siphonous, coenocytic cells. Cells 506.76 ± 34.22 μm in diameter, divided into an inner medullar mesh of small, intertwined siphons (diameter 261.76 ± 29.18 μm), and utricles (length 112.00 ± 11.60 μm). Medullar siphons width of 3.06 ± 1.13 μm. Utricles tightly packed and clavate to cylindrical, with tips rounded and smooth, lacking hairs. External utricle tip morphology consistent within and across specimens. Tip wall thickness (12.38 ± 2.87 μm) about four times that of utricle lateral wall (2.90 ± 0.59 μm). Contact between utricle tips is roughly pentagonal to hexagonal. No gametangia can be observed. At their base, utricles connect to the medulla via a single medial siphon branching basally into two other siphons.

## Discussion

The Neoproterozoic diversification of green algae has been regarded as a key event following the earlier radiation of the red algae sister group, which was tied to the use of different developmental strategies, namely multicellularity and large siphonous cells, upon which further speciation could have been promoted by selective pressures associated with global glaciations [7]. In this context, separate time-calibrated molecular analyses placed the origin of *Codium* either during the early Paleozoic or the late Mesozoic and that of all Bryopsidales in or close to the Cambrian [7, 9]. Worth noting is that the fossil evidence used for calibration before the Phanerozoic has been scarce to non-existent, largely because of taxonomic uncertainties. Del Cortona and colleagues [7] recognized in particular the morphological affinities of the late Tonian *Proterocladus* with Cladophorales but considered the data too much at odds with their model. The very recent integration of *Proterocladus* for time calibration of a phylotranscriptomic analysis, however, supported a Cambrian origination of Bryopsidales [31]. The presence of an at least stem group *Codium* in the Ediacaran, as documented here, imposes a revised timing of algal radiation events and in particular an older origination and diversification for at least Chlorophyta. Considering that the origin of *Codium* would be at least about 100 million years older than the closest molecular estimates [7], this could also apply to green seaweeds as a whole. Among green algae, multicellular and siphonous strategies as well as related diversifications inside Ulvophyceae therefore arguably predated Cryogenian glaciation events. This finding is broadly consistent with other pieces of paleontological evidence suggesting a much earlier origin and diversification of chlorophytes [3].

The resolution of a small spherical form of *Codium* as stem group candidate aligns with phylogenetic predictions optimizing the smallest and unbranched morphotypes as ancestral for the genus [23]. Our finding would confirm that the plesiomorphic condition is spherical for *Codium*, from which mat-forming taxa were first to diverge. By contrast, our fossil conflicts with an optimization of composite utricles being ancestral, pointing to simple utricles being the plesiomorphic condition instead. *Protocodium* further suggests that the ancestral developmental condition of utricles is to branch out at their base via a single medullar filament.

**Fig. 5:**
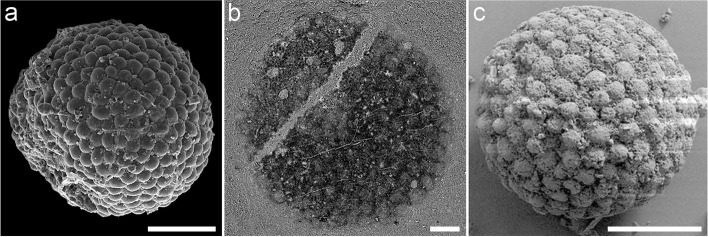
Similarity in external morphology between *Codium*, *Chuaria*, and *Megasphaera* at the *Megaclonophycus* stage. **a** Bryopsidales alga *Protocodium sinense* gen. et sp. nov., holotype NWULJG 10,034, scanning electron microscopy (SEM). **b** “Multicellular aggregate” form of *Chuaria circularis*, backscattered-electron SEM at 25 keV, from the Tonian Liulaobei Formation. Modified from Tang et al. [18]. **c** *Megaclonophycus* stage of the *Megasphaera inornata* embryo from the Ediacaran Weng’an biota of the Doushantuo Formation. Preserved without enclosing envelope. Note that only early *Megaclonophycus* developmental stages of *Megasphaera*, such as the one represented here, which possess the adequate number of cells, are directly comparable to *Protocodium* and *Chuaria*. Modified from Xiao et al. [13]. Contrary to the embryo in **c**, the form of *Chuaria* in b cannot yet be accurately distinguished from Protocodium based on internal anatomy. **b**, **c** Images courtesy of Qing Tang, Shuhai Xiao and Xunlai Yuan. Scale bars: 200 μm

Our fossils potentially also shed light on the identity of the common and cosmopolitan *Chuaria*, or at least certain representatives of this wide-ranging and poorly constrained taxon [16]. Fossils attributed to *Chuaria* are circular to ovate and common worldwide in Precambrian sediments, but their affinity has long remained uncertain [16, 32]. Membranous elements interpreted as cell walls [32] characterizing the type species, *C. circularis*, have led authors to attempt to circumscribe the genus to a vesicular, “acritarch” form [16]. However, external aspects described as characteristic of multicellularity in some other morphs with particularly detailed preservation [18] are indistinguishable from what we reveal here to be differentiated algal cells close to *Codium*, that is, spherical with an outer layer of utricles (Fig. 5). Furthermore, smaller specimens of the “multicellular” *Chuaria* aggregates are only about twice as large as those of *Protocodium* [18]. In contrast to “Doushantuo” embryos, which can be readily distinguished by their internal structure, the internal morphology associated with the “compound” aspect of *Chuaria* has not yet been resolved (Fig. 5). A multicellular interpretation would be supported by vesicular specimens preserving in the form of a cuticular envelop, sometimes torn on one side, but some other similar forms such as *Suketea* also preserve central rings reminiscent of a medulla [33]. It was argued that the co-occurrence of both vesicular and “multicellular” forms of comparable sizes supported their interpretation as parts of the same organism [18], but neither can we rule out the co-existence of similar-sized spherical taxa, as embryonic forms illustrate (Fig. 5). These apparent contradictions call for a reevaluation of morphological diversity within *Chuaria*, and correlatively question the prevalence and significance of its association with *Tawuia* [33]. Consequently, the discovery of *Protocodium sinense* could imply that *Codium* or *Codium*-like taxa were in fact common worldwide even since before the Tonian.

## Conclusions

Detailed external and internal three-dimensional preservation of fossils at the terminal Ediacaran Dengying Formation allowed us to constrain the taxonomic identity of micrometric spherical, “compound” eukaryotes as close relatives to *Codium*, which we helped approximate via the use of phylomorphospace reconstruction. Although we formally recognize the morphotype as stem group largely based on a size difference, extensive morphological similarities make it reasonable to use *Protocodium* as minimal calibration point for the origin of *Codium*, or more conservatively that of Bryopsidales. This new data point is expected to have a decisive impact on future molecule-based timing of chlorophyte evolution. Such timing may be further altered substantially, should “*Chuaria*” be recognized as encompassing separate algal forms that may relate to *Protocodium* or other overlooked members of extant chlorophyte relatives. Finally, *Protocodium* can serve as a clear example of extreme longevity of a eukaryote cell plan with moderate phenotypic differentiation.

## Methods

### Geologic background

The fossil material documented here was collected from the intraclastic dolostone of the Beiwan Member, Dengying Formation at the Lijiagou section in southern Shaanxi Province, China, which is located in the northwestern margin of the Yangtze Platform [34]. The Dengying Formation in this area is conformably underlain by the Doushantuo Formation and unconformably overlain by the basal Cambrian Kuanchuanpu Formation (Bureau of Geology and Mineral Resources of Shaanxi Province, 1989). At the studied section, the Dengying Formation (551–538 Ma) consists of three lithographic units: the lower Algal Dolostone Member, composed of thick-bedded dolostone, the middle Gaojiashan Member, consisting of thin-bedded calcareous mudstones and siltstones, and the upper Beiwan Member, dominated by thick-bedded intraclastic dolostone [35, 36] (Fig. 1). The five fossil specimens studied here were extracted from a total 500 kg of rock sample. Co-occurring taxa include tubular fossils *Cloudina hartmanae*, *C. sinensis*, *C. ningqiangensis*, *C. lijiagouensis*, *C. xuanjiangpingensis*, a *Cloudina*-like fossil, *Multiconotubus chinensis*, *Sinotubulities baimatuoensis*, *S. triangularis*, *S. pentacarinalis*, and *S. hexagonus*, cyanobacteria fossils *Girvanella*, *Obruchevella*, *Cambricodium* and *Epiphyton*. Vase-shaped microfossils (VSMs) and other problematic forms were collected from the same fossiliferous horizon [34, 37]. Cyanobacteria [37] and yet undescribed algae account for approximately 20% of this remaining material. A detailed sedimentological investigation on the Dengying Formation at the Liajiagou section is lacking to this date, but abundant coarse, intraclastic dolostone underlying pure dolostone (Additional file 5: Fig. S4) suggests a depositional environment of the Beiwan Member characterized by shallow water, and, in light of the whole sequence (Fig. 1d), part of a shallowing upward cycle. The material is currently deposited at the Shaanxi Key Laboratory of Early Life and Environments (LELE) and Department of Geology, Northwest University (NWU), Xi’an, China.

### Preparation and observation

All five specimens were collected from 10 g sample residues dissolved in 5–10% acetic acid. A FEI Quanta 450 scanning electron microscopy (SEM) was carried out to investigate the microstructure on the surface of the fossils. Measurements were made from digital photographs using Photoshop CC 20.0.4 and ImageJ v.2.5.0. Full morphometrics were possible for only four specimens (Additional file 1: Dataset S1). The chemical measurements of minerals were analyzed by an electron probe microanalyzer (EPMA) (JXA-8530F Plus, JEOL, JP) equipped with five wavelength dispersive spectrometers. X-ray profiles and quantification were carried out at 30 kV. Computed X-Ray microtomography (Micro-CT) was used to reveal the internal structures of the specimens. Specimen scanning was performed by Zeiss X-radia 520 Versa. Each scan generated a set of radiographs saved as TIFF stacks which were further processed with the DRAGONFLY 4.1 software (http://www.theobjects.com).

### Multivariate analysis

Character list, data, and tree topology were taken from Verbruggen et al. [23] (Additional file 1: Dataset 1). Analytical operations were performed in R mainly using the packages *ape*, *cluster*, *phytools*, *RColorBrewer*, and *vegan*. Neighbor-joining, UPGMA, and WPGMA clustering methods were based on Gower dissimilarities calculated with the function *daisy* of the package *cluster*, treating variables as either categorical (nominal) or numeric, as relevant. A principal coordinate analysis (PCoA) was then performed based on the same dissimilarity matrix. A “broken stick” test determined that only the first two dimensions were significant in explaining total variance, with dimensions 3 and 4 close to significance at 95%. A k-means clustering method failed to find significance for up to 10 groups, and we therefore chose to display the solution found for seven groups, based on its correspondence with the graphical result. Loadings were computed as chi-square *p*-values from a symmetrical subdivision of the dataset into four regions, after Foote [38], and were represented as pie plots according to significance at 95%, using the empirical formula (0.05-(x/0.05))/0.05 (Additional file 1: Dataset S1). The phylomorphospace was created using a projection of the main phylogram in Verbruggen et al. [23].

## Supplementary Information

**Additional file 1: Dataset S1.** *Protocodium* morphometrics, morphological matrix from Verbruggen et al. (2007) including *Protocodium*, and relative loading p-values of the PCoA analysis based on a significance at 95%.

**Additional file 2: Figure S1.** *Protocodium sinense* from the Dengying Formation of South China. a, b Scanning electron microscopy of the fossil surface. a NWULJG 10021. b NWULJG 10015. Scale bars, 100 μm.

**Additional file 3: Figure S2.** EPMA analysis of a *Protocodium sinense. *Specimen NWULJG 10042, elements as indicated. Scale bar: 100 μm.

**Additional file 4: Figure S3.** Hierarchical clustering of *Codium* morphotypes based on morphology. a Neighbour-Joining. b UPGMA. c WPGMA.

**Additional file 5: Figure S4.** Petrographic observations of dolostones in the Beiwan Member, Dengying Formation, at the Lijiagou section. a Field photo of sample BW-26. b Petrographic photograph of sample BW-26 showing dolostone with abundant intraclast. c Field photo of sample BW-57. d Petrographic photograph of sample BW-57 showing pure dolostone. PPL–plane polarized light.

## Data Availability

All data used for this study are made available as supplementary material. Fossil collection followed all relevant Chinese regulations. Type material (holotype NWULJG 10,034 and paratypes NWULJG 10,015, 10,021, 10,026, 10,042) deposited at Shaanxi Key Laboratory of Early Life and Environments (LELE) and Department of Geology, Northwest University (NWU), Xi’an, China, and accessible upon request.
