# Supplementary figures and images for: A stem group Codium alga from the latest Ediacaran of South China provides taxonomic insight into the early diversification of the plant kingdom
Source: BMC Biol. 2022 Sep 21;20:199. doi: 10.1186/s12915-022-01394-0 (PMC9491005; doi:10.1186/s12915-022-01394-0)

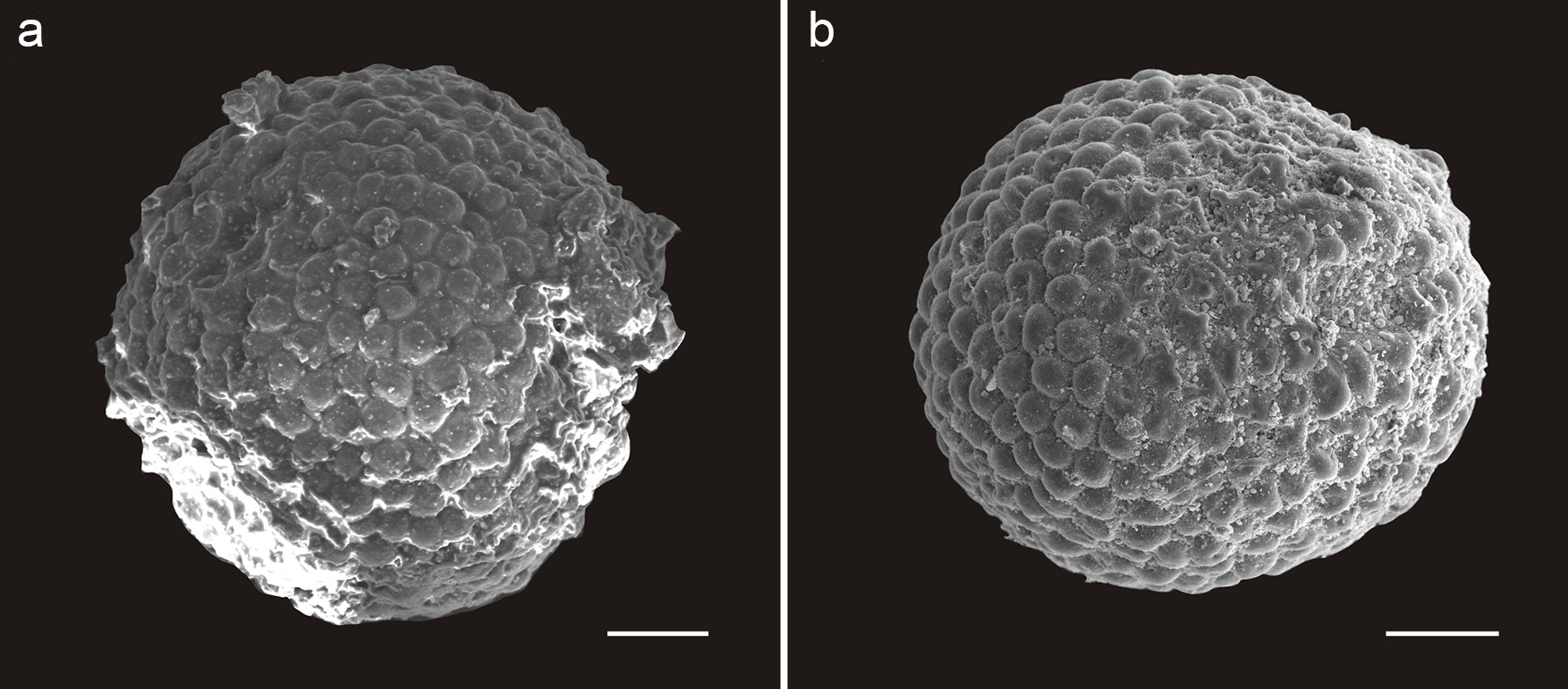

Supplement: Supplementary file 2 — Additional file 2: Figure S1. Protocodium sinense from the Dengying Formation of South China. a, b Scanning electron microscopy of the fossil surface. a NWULJG 10021. b NWULJG 10015. Scale bars, 100 μm. [file 12915_2022_1394_MOESM2_ESM.tif]

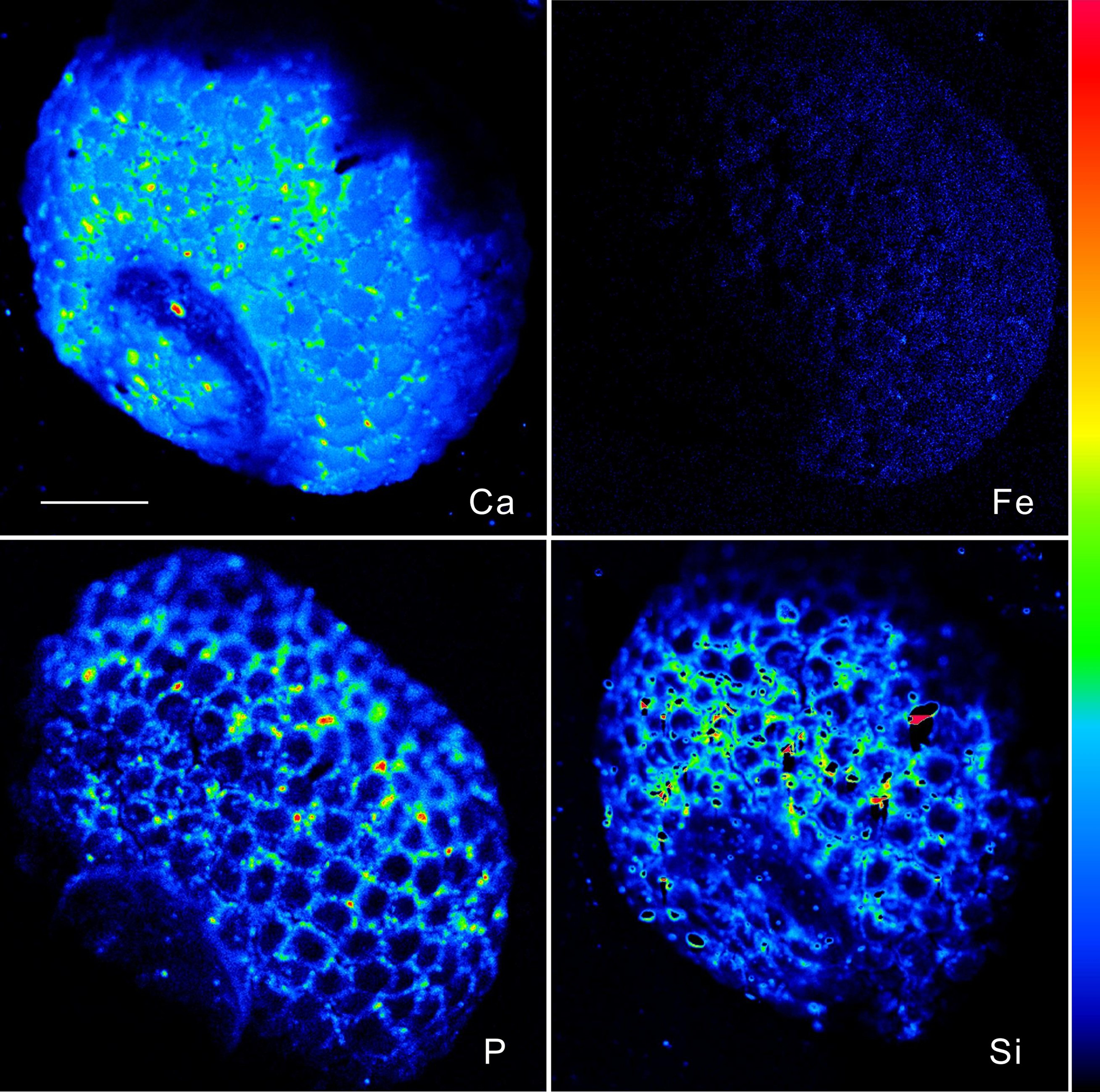

Supplement: Supplementary file 3 — Additional file 3: Figure S2. EPMA analysis of a Protocodium sinense. Specimen NWULJG 10042, elements as indicated. Scale bar: 100 μm. [file 12915_2022_1394_MOESM3_ESM.tif]

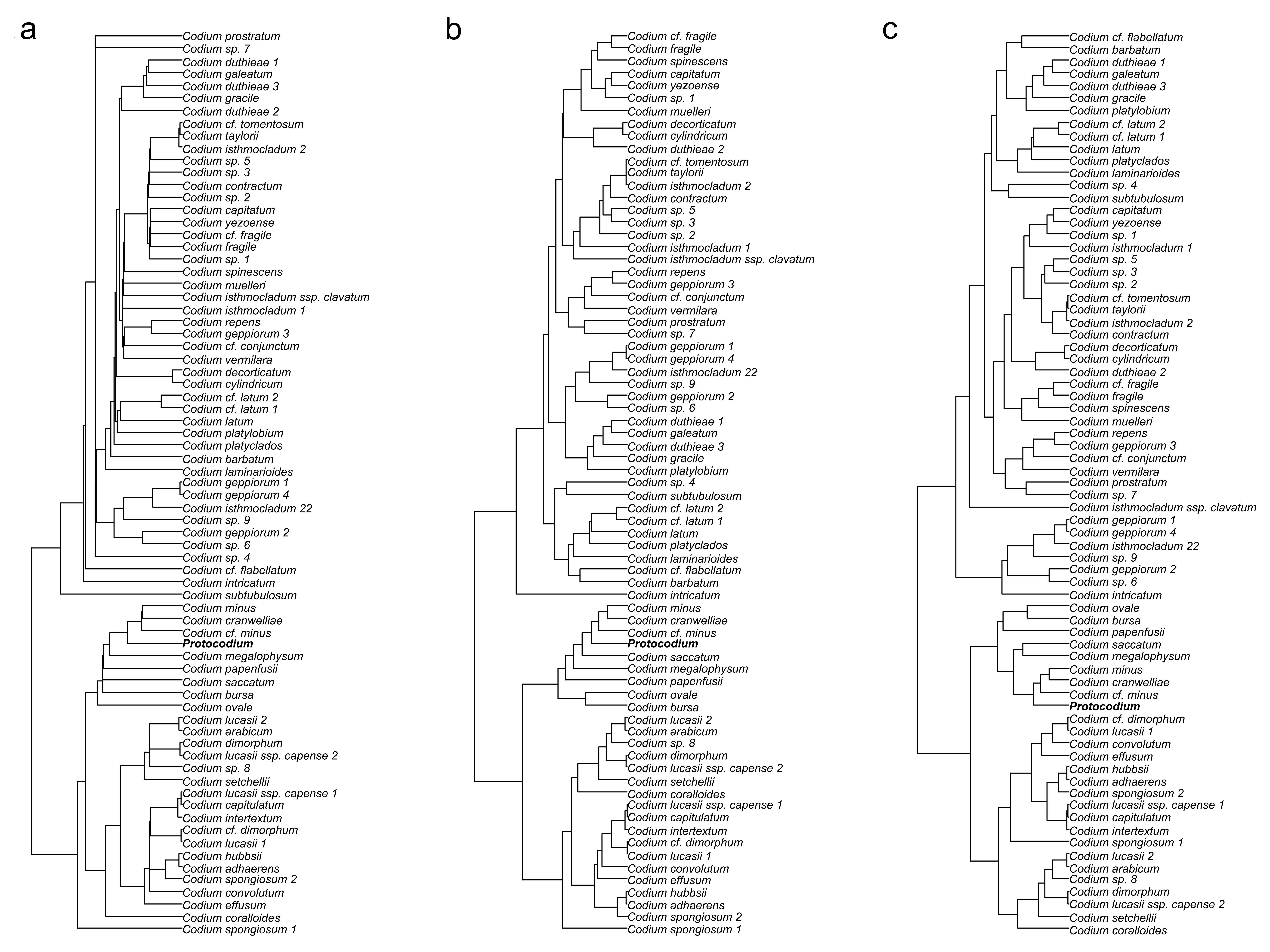

Supplement: Supplementary file 4 — Additional file 4: Figure S3. Hierarchical clustering of Codium morphotypes based on morphology. a Neighbour-Joining. b UPGMA. c WPGMA. [file 12915_2022_1394_MOESM4_ESM.tif]

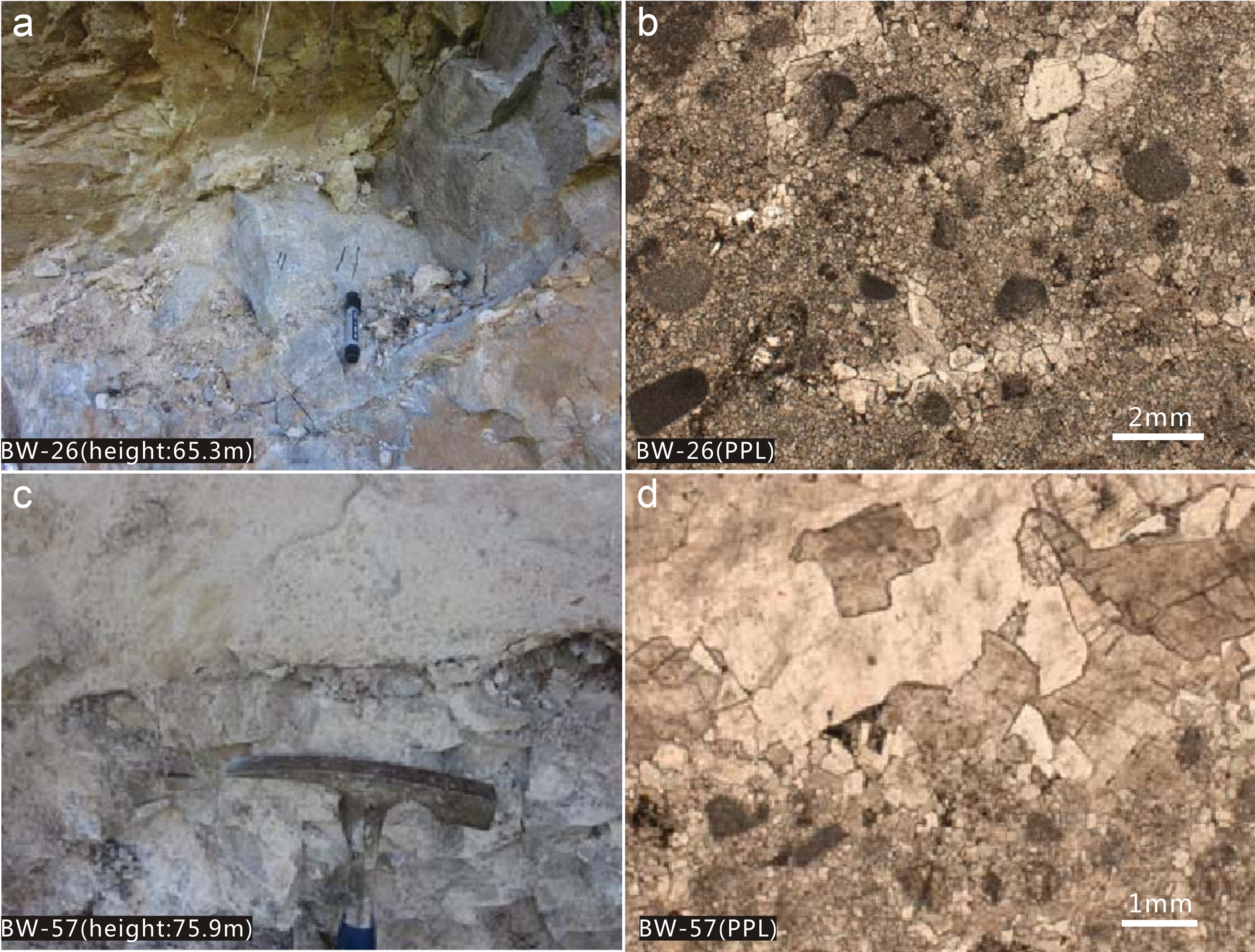

Supplement: Supplementary file 5 — Additional file 5: Figure S4. Petrographic observations of dolostones in the Beiwan Member, Dengying Formation, at the Lijiagou section. a Field photo of sample BW-26. b Petrographic photograph of sample BW-26 showing dolostone with abundant intraclast. c Field photo of sample BW-57. d Petrographic photograph of sample BW-57 showing pure dolostone. PPL–plane polarized light. [file 12915_2022_1394_MOESM5_ESM.tif]
